# The Influence of Anthropometric Indices and Intermediary Determinants of Hypertension in Bangladesh

**DOI:** 10.3390/ijerph18115646

**Published:** 2021-05-25

**Authors:** Sally Sonia Simmons, John Elvis Hagan Jr., Thomas Schack

**Affiliations:** 1 Department of Social Policy, London School of Economics and Political Science, Houghton St, London WC2A 2AE, UK; ssimmons@edu.hse.ru; 2 Institute of Demography, National Research University-Higher School of Economics, 101000 Moscow, Russia; 3 Department of Health, Physical Education & Recreation, College of Education Studies, University of Cape Coast, Cape Coast PMB TF0494, Ghana; 4 Neurocognition and Action Research Group- Biomechanics, Faculty of Psychology & Sport Sciences/CITEC, Bielefeld University, 33501 Bielefeld, Germany; thomas.schack@uni-bielefeld.de

**Keywords:** anthropometric indices, Bangladesh, body mass index, hypertension, waist circumference, waist-to-height ratio, waist-to-hip ratio

## Abstract

Hypertension is a major public health burden in Bangladesh. However, studies considering the underlying multifaceted risk factors of this health condition are sparse. The present study concurrently examines anthropometric parameters and intermediary factors influencing hypertension risk in Bangladesh. Using the 2018 World Health Organisation (WHO) STEPwise approach to non-communicable disease risk factor surveillance (STEPS) study conducted in Bangladesh and involving 8019 nationally representative adult respondents, bivariate and multivariate logistic regression analyses were performed to determine the association between anthropometrics, other intermediary factors and hypertension. The regression results were presented using the odds ratio (OR) and adjusted odds ratio (AOR) at 95% confidence intervals (CIs). The risk of hypertension was higher among females and males who were 40 years and older. However, among females, those who were age 60 years and older were more than twice and thrice more likely to be hypertensive compared to those in the younger age groups (18–39, 40–59). Females who were obese (body mass index [BMI], waist to hip ratio [WHR], waist to height ratio [WHtR]) or had high waist circumference [WC] were twice as likely to be hypertensive. Males and females who were physically active, consuming more fruits and vegetables daily and educated had lower odds of developing hypertension. Key findings suggest that the association between anthropometric indices (body mass index [BMI], waist to hip ratio [WHR], waist to height ratio [WHtR]), waist circumference [WC]), other intermediary determinants (e.g., education, physical activity) and hypertension exist across gender and with increasing age among adults in Bangladesh. Developing appropriate public health interventions (e.g., regular assessment of anthropometric parameters) for early identification of the risk and pattern of hypertension through appropriate screening and diagnosis is required to meet the specific health needs of the adult Bangladesh population.

## 1. Introduction

Hypertension is a risk factor for the pathogenesis of cardiovascular diseases [1]. About 53% of all cardiovascular diseases related deaths are due to complications of hypertension [2]. Hypertension accounts for half and two-thirds of ischaemic heart disease and stroke cases and 7.6 million premature deaths [3,4]. Available evidence suggests that global prevalence of hypertension will rise from 26% in 2000 to 29.2% by 2025 [5]. Although hypertension is more acute in higher-income counties such as the USA [6], its prevalence is rapidly increasing in the low and middle-income countries (LMICs) [7], with countries in Asia, specifically South Asia, confronted with an increasing burden [8]. Reaching a hypertensive status depends on blood pressure measurement outcomes (i.e., systolic blood pressure of ≤120 mmHg- normal and ≥140 mmHg- high or diastolic blood pressure of ≤80 mm Hg- normal and ≥90 mmHg- high) [3].

Recent socio-economic development related to urbanization, improved life expectancy, unhealthy dietary practices (e.g., consumption of saturated fat and red meat), and lifestyle changes have increased the prevalence rates of hypertension, particularly in Bangladesh [9,10,11]. Hypertension is reported to be associated with an increased risk of stroke, myocardial infarction and other cardiovascular diseases (CVDs) in Bangladesh [12]. About 12 million (18%) of Bangladeshi adults aged 25 and above are hypertensive [3]. More females (56.2%) than males (43.8%) are hypertensive [13] and poorly manage high blood pressure (BP) [14]. Shifts in nutrition increase overnutrition in Bangladesh [15] as the excessive intake of dietary macro- and micronutrients, overnutrition, alters body fat composition [16]. Body fat is a common risk factor for cardiometabolic diseases [17] and measured using anthropometric measures such as waist circumference, hip circumference (HC), waist-to-hip ratio (WHR), body mass index (BMI), and waist-to-height ratio (WHtR) [18].

The social determinants of the health framework show that two broad factors (i.e., structural and intermediary) are associated with health outcomes. In the context of Bangladesh, structural factors may include public policies implemented at macro and micro levels to influence health outcomes, including hypertension risk [15]. Additionally, intermediary factors, including immutable determinants (e.g., sex, age, residence, ethnicity) and other modifiable factors such as perceived disease risk, lifestyle changes [e.g., dietary habits, physical activities, increased automation, alcohol consumption] [19], and material circumstances [e.g., income, type of job, employment] have also been cited as attributable to hypertension [20].

While the trend of hypertension burden in Bangladesh is known [4,18], research on the effects of anthropometrics, together with intermediary health determinants on hypertension, is an emerging area. The inclusion of multiple anthropometric parameters as part of disease risk detection protocols in Bangladesh can contribute to screening individuals at greater risk of hypertension in the country. Early identification of the most at-risk groups would help with timely referrals to well-structured health services for monitoring and management [21]. The present study examines anthropometrics and other intermediary factors influencing hypertension risk among males and females in Bangladesh. 

## 2. Materials and Methods

### 2.1. Data Source 

The World Health Organisation (WHO) STEPwise approach to non-communicable disease risk factor surveillance (STEPS) 2018 study conducted in Bangladesh is the source of data for the study. STEPS 2018 is the second wave of STEPS longitudinal studies in Bangladesh focusing on six main areas of non-communicable diseases (NCDs) risk surveillance; demographics, diseases, health, lifestyle, physical, and biochemical measurements among adults aged 18–69. A nationally representative sample of 8185 participants was recruited for the survey. The Bangladesh Bureau of Statistics (BBS) developed the primary sampling units (PSUs) using the multistage and geographically stratified probability-based sampling methods. The participants of the study were selected from six administrative units: Barisal; Chittagong; Dhaka, Khulna; Rajshahi; Sylhet. Each administrative unit was subdivided into districts (Zilla) and sub-districts (Upzilla). Upzillas were further divided into mauzas (rural stratum) and mahallas (urban stratum), the PSUs for the study’s sampling methods. 400 PSUs (200 mauzas and 200 mahallas) were selected. Data were collected from November 2017 to April 2018. A detailed description of the Bangladesh STEPS study’s methods and materials, including the data and data collection procedures, is provided on the project website [22,23,24].

### 2.2. Study Variables

The outcome variable for the study was hypertension, with a “yes” or “no’ response to the question “Have you ever been told by a doctor or other health worker that you have raised blood pressure or hypertension?” Overall, twenty variables were selected for the study. These variables were sociodemographic profiles (i.e., age, sex, residence and level of education), anthropometrics (i.e., weight, height, hip circumference, waist circumference), economic factors (i.e., employment), and health (i.e., hypertension, systolic and diastolic blood pressure, and heart rate (beats per minute)/pulse rate, antihypertensive medication), and behavioural variables (i.e., physical activity, fruits intake, vegetable intake, tobacco use, and alcohol consumption). These variables were selected to achieve numerically stable and adaptable models [25]. Age, height, weight, hip circumference, waist circumference, systolic and diastolic blood pressure, and heart rate (beats per minute)/pulse rate were numeric variables. Sex, employment, tobacco use, alcohol consumption, antihypertension medication intake, and hypertension were dichotomous variables, and level of education was a polychotomous variable.

Age was transformed into a categorical variable to reflect significant stages of ageing (i.e., 18–39, 40–59, 60+) [26]. Level of education was categorised as no education, primary, secondary and tertiary. Systolic and diastolic blood pressure was transformed into a categorical variable, with three elements (i.e., normal BP, prehypertension [moderate BP], hypertension [high BP] based on the WHO International Society of Hypertension criterion [27]. Normal systolic BP was <120 mmHg and diastolic BP was <80 mmHg; prehypertension status or moderate systolic BP was 120–139 mmHg, and diastolic BP was 80–89 mmHg; high systolic BP or hypertension was ≥140 mmHg, and diastolic BP was ≥90 mmHg.

Waist circumference was categorised as low (males: <94 cm, females: <80 cm); high (males: 94–102 cm, females: 80–88 cm); very high (males: >102 cm, females: >88 cm). Height, weight, hip circumference and waist circumference were used to estimate other anthropometric measures (i.e., BMI, WHR, BAI, and WHtR). BMI was measured as the ratio of weight (in kilograms) to height (in metres). This derivative was estimated based on WHO standard recommendations for BMI (<18.5 kg/m^2^ underweight; 18.5–24.9 kg/m^2^—normal; 25.0–29.9 kg/m^2^—overweight; ≥30.0 kg/m^2^—obese) [28]. WHR was estimated as the ratio of waist to hip circumference and categorised as low (0.80 or lower for females; 0.95 or lower-for males), moderate (0.81–0.85 for females; 0.96–1.0 for males), and high (0.86 or higher for females; 1.0 or higher for males) [29]. WHtR was defined as the ratio of waist circumference to height and categorised as slim (0.42 or lower for females; 0.43 or lower-for males), normal (0.43–0.48 for females; 0.44–0.51 for males), overweight (0.49–0.53 for females; 0.52–0.56 for males) and obese (0.54 or higher for females; 0.57 or higher for males). These body fat composition measures were introduced to evaluate the influence of body fat on health [30]. Fruit and vegetable intake were combined to create a new variable, fruits and vegetables intake. The newly created variable was categorised as <2 servings per day, 2–4 servings per day and ≥5 servings per day. All domains of physical activity (PA), including transportation (walking/biking), work and leisure-time/recreational activity, and time spent (converted into minutes) on PA, were used to calculate metabolic equivalent task (MET)-min per week. A MET-min of <600 per week was coded as low PA, 600–3000 MET-min as moderate PA, and >3000 MET-min as high PA [4,31]. All females who were pregnant at the time of the survey were omitted from the sample under study. The final sample used for the study was 8019. Missing values for the variables were imputed using the predictive mean matching multiple imputation approach to help reduce the effects of missing data in all computations [32].

### 2.3. Data Analyses 

Selected sociodemographic and other variables were summarised and stratified by sex to provide a clearer understanding of the distribution among males and females. The distribution was complemented with a test of variation in the outcome. The prevalence and 95% CIs of pulse rate, normal, prehypertension, hypertension, and antihypertensive drug usage were estimated among males and females. Multiple logistic regression models were built to ascertain how each of the predictors contributed to predicting the value of the response variable (hypertension) [33]. Four models (two for males and females) were fitted for this purpose. Statistical significance tests were based on a two-sided probability set at *p* < 0.05. All analyses were computed in R statistical software version 4.04 (R Foundation for Statistical Computing, Vienna, Austria). R programme is a software environment and programming language designed for statistical analysis, graphics representation and reporting. The mice, survey, dplyr and extraoperators packages in R were used for the current study [34,35,36,37]. 

## 3. Results

### 3.1. Descriptive Analysis on the Distributions of Sociodemographic and Anthropometric Characteristics of Males and Females

Table 1 shows the distributions of all sociodemographic and anthropometric characteristics of males and females. A total of 3804 (47%) males and 4215 (53%) females aged 18 or older (total of 8019) participated in the study. Among the 3804 males, 1875 (49.3%) were aged 18–39 years, 1919 (50.4%) were of rural residence, 1718 (45.2%) had no education, 2650 (69.7%) had 2–4 servings of fruits and vegetables per day, 2322 (61.0%) had moderate physical activity (600–3000 MET-min per week), 715 (18.8%) were obese (BMI), 557 (14.6%) were obese (WHtR) and 3091 (81.3%) were not hypertensive. Among the 4215 females, 2491 (59.1%) were aged 18–39 years, 2187 (51.9%) were of rural residence, 1933 (45.9%) had no education, 2679 (63.6%) had 2–4 servings of fruits and vegetables per day, 2587 (61.4%) had moderate physical activity (600–3000 MET-min per week), 1142 (27.1%) were obese (BMI), 1897 (45.0%) were obese (WHtR), and 3231 (76.7%) were not hypertensive.

Table 2 shows the prevalence and 95% CI of pulse rate, normal blood pressure (BP), prehypertension, hypertension and intake of anti-hypertension medications among males and females in Bangladesh. The distribution of the systolic blood pressure shows that females had high blood pressure (i.e., were hypertensive) 8.72%, (0.15–0.20) and normal systolic blood pressure 24.00, (0.46–0.51) as compared to 7.14% (0.12–0.16) and 19.82% (0.37–0.42) had prehypertension in males. Many females [34.03%, (0.66–0.71)] than males [31.5%, (0.60–0.65)] used antihypertensive medications.

### 3.2. Multivariate Logistic Regression Analyses on Anthropometric, Sociodemographic and Behavioural Factors Associated with Hypertension

Multivariate logistic regression outcomes for anthropometric, sociodemographic and behavioural factors associated with hypertension are presented in Table 3. The risk of hypertension was higher among females and males who were 40 years and older. Among females, those who were aged 60 years and older were more than twice and thrice (OR = 2.90, 95% CI = 2.43–3.73; AOR = 4.71, 95% CI = 3.87–6.27) as likely to be hypertensive as compared to those in the younger age groups (18–39, 40–59). Among males, persons age 60 years and older were more likely (OR = 3.42, 95% CI = 2.65–4.73; AOR = 3.78, 95% CI = 2.76–5.17) to be hypertensive than any other age cohort. Females who were obese were more likely to be hypertensive as compared to those who were slim, underweight or had moderate body fat levels. Males and females who were physically active (females [OR = 0.42, 0.33–0.54; AOR = 0.47, 0.39–0.64; males [OR = 0.51, 0.42–0.66; AOR = 0.67, 0.62–0.79]), not consuming alcohol, and had some form of education had lower odds of hypertension.

## 4. Discussion

The present study assessed the distribution of hypertension and possible factors influencing hypertension in Bangladesh. The study revealed that the prevalence of hypertension in Bangladesh was about 21.2%: 23.3% among females and 18.7% among males. This finding is similar to a cross-sectional rural study in Bangladesh that found that about 18.8% of Bangladeshi males were hypertensive [31]. Contrarily, the prevalence of hypertension among females reported is relatively lower than the 56.2% reported by Hanif [13]. Also, the overall prevalence of obesity (BMI) was 23.2%; 27.1% females and 18.8% males. The prevalence of obesity documented in the present study is lower than findings from the studies conducted by Islam et al., [38] and Siddiquee et al., [39], who reported 28% and 26.2%, respectively. The reasons for the disparities are open to speculation. Possibly, the differences in sampling, including sizes, methodological variations, BMI cut-off criteria and population of interest might be the underlying reasons. For instance, the variations between studies could be attributed to the distinctions in the body composition measurements and cut-offs between populations and among different population groups in the same country [40], especially in countries like Bangladesh where there seem to be large sub-regional dimensions [41]. These inconsistencies reinforce the need to assess the severity of hypertension and its attributable factors among the most at-risk populations within a specific context. The prevalence of abdominal obesity, measured as WHR, among females was 49.1%. This finding is similar to the 49% and 48.7% reported by previous studies in Bangladesh [38,39]. The higher prevalence of abdominal obesity among females indicates that using a single index, BMI in particular, may lead to the exclusion and misclassification of many people who are obese [38,42]. In addition, this finding affirms the advocacy of WC, WHR and WHtR as complements of BMI when assessing disease risk [43].

The distribution of systolic blood pressure, diastolic blood pressure and pulse rate varied among males and females. Females maintained normal pulse and were more hypertensive than males. This might have been a population effect (i.e., the inclusion of women who have ended their reproductive years) as women, after reaching menopause, experience an increase in blood pressure irrespective of ethnicity [44]. Hormonal changes during menopause predispose women to obesity, increased blood pressure, and dietary salt sensitivity and cause higher blood pressure [45]. Also, more females than males were taking antihypertensive medications. Women are more conscious of their health and take greater responsibility than men on health behaviours and the potential impact on illness; thus, they might take these antihypertensive drugs as a precautionary measure against the risk of developing hypertension and other co-morbidities [46,47,48]. While the prevalence of hypertensive cases is similar to most research evidence in Bangladesh [2,23], all risk factors showed a greater risk of hypertension among males than females. The current finding confirms existing studies which found increased hypertension and other cardiovascular diseases risk among males than females [31,49,50]. 

The association between WC, BMI, WHtR, age, physical activity, alcohol consumption, and hypertension is similar to but not entirely in unison with several studies in Bangladesh and elsewhere [3,15,20,51,52]. Males and females who were 60 years and older and those with higher BMI, WHR, WHtR and WC were about 2 to 3 times more likely to be hypertensive. Higher BMI, WHR, WHtR and WC result from increased fatty tissues; which can increase arterial and vascular stiffness and affect how the heart pumps blood [53]. The association between WC, BMI, WHR, WHtR and hypertension corroborate previous research that revealed that these anthropometric indices are essential predictors of cardiometabolic conditions across females [54,55,56,57] and males [54,58]. These linkages show the significance of identifying the pattern of the body fat distribution through these anthropometric indices and the control of excess body fat-obesity and high WC as part of effective hypertension management procedures among the adult population in Bangladesh [41]. The distinct sex disparities in hypertension risk in the older years of life found in the present study confirm findings from previous studies [51,59]. Hypertension increases as a function of age through biological and social pathways [31,60,61]. Ageing alters the vascular system structure and functioning. There are changes in the arteries elastic tissues, causing artery stiffness, less compliance to blood flow and, therefore, raise blood pressure [31,60]. The elderly in many contexts, including Bangladesh, are often lonely and isolate from many social activities. Loneliness leads to detrimental physiological and health problems like depression, increased cardiometabolic dysfunction and high blood pressure [61]. Physical activity is widely known to reduce hypertension risk [31,62,63,64,65]. Regular exercise (i.e, walking, cycling) has been shown to lower blood pressure and forms one of the most clinically recommended hypertension management and treatment. Physical activity has an immediate post-exercise hypotension effect and can persist for longer periods. Continuous engagement in physical activities may reduce existing pressure levels to normal [64]. 

It was also observed that modifiable determinants such as not consuming alcohol, increased fruits and vegetables intake and higher education lowered the risk of hypertension among females and males. Several studies have proven that unhealthy behaviours (e.g., inadequate fruits and vegetable consumption, low physical activity, binge drinking) increase cardiovascular diseases across varied age groups and diverse populations [66,67,68,69]. Hence, regular advocacy on healthy lifestyles could help reduce or delay hypertension risk and its associated comorbidities in Bangladesh.

### 4.1. Strengths and Limitations

The study’s strengths were the use of a large nationally representative sample and the concurrent assessment of anthropometric measures and other multifaceted determinants of hypertension in Bangladesh. The World Health Organisation (WHO) STEPwise approach to non-communicable disease risk factor surveillance (STEPS) protocol uses high standards and valid data collection procedures. Hence, measurement errors and biases are limited compared to other cross-sectional studies. Despite these strengths, the present study has some weaknesses. The main weakness is the cross-sectional design employed, implying that causality cannot be ascribed to the noted outcomes; hence identified associations can only be inferred. Due to the self-reported nature of the design, social desirability and recall bias-related issues could in part influence the current findings. Another weakness of the study is the exclusion of the influence of biomarkers or biochemical measures of hypertension. Further study designs, involving the use of biomarkers or biochemical measures, will provide insight into the risk of hypertension in Bangladesh. Notwithstanding these limitations, our observations provide insights into some of the possible effects of anthropometrics and intermediary determinants of health on hypertension. 

### 4.2. Practical Implications

The critical implications of the present study are the increased burden of hypertension among males and females in Bangladesh, with persons who are 60 years and older, and the female population being 2–3 times more likely to suffer hypertension in the country. A positive association between increasing age and hypertension was also observed. Because age is immutable and life expectancy in Bangladesh is rising, demographic opportunities will increase [8]. This trend will subsequently increase the risk of hypertension among the ageing population in Bangladesh [70]. The overnutrition, obesity or anthropometric indices such as BMI, WHR, WC, and WHtR predicted hypertension in Bangladesh. Hence, behaviour modification should be prioritised in the country. Modifiable interventions such as body weight reduction through regular physical activity and improvement of dietary practices (e.g., reducing the intake of high dense energy or fatty foods) are encouraged across the population. Again, because hypertension is relatively high in Bangladesh, the healthcare system in the country requires pragmatic interventions such as early identification through screening and diagnosis of the disease across rural and urban settlements in the country. 

## 5. Conclusions

Findings suggest that sex and increasing age differences exist in the association between anthropometric indices (BMI, WHR, WC, and WHtR) and intermediary determinants (e.g., education, physical activity) of hypertension among adults in Bangladesh. These anthropometric parameters should be assessed periodically among male and female adults in Bangladesh to mark the risk of hypertension in the country. Developing effective public health interventions for the early identification of the risk and patterns of hypertension through appropriate screening and diagnosis are needed to meet the specific needs of the Bangladesh population. Other intervention programmes should target behaviour modification (e.g., adherence to regular physical activity and appropriate dietary consumption) at the population level to help minimise the burden of hypertension in the country. Regular sensitisation campaigns are encouraged to promote primary prevention and secondary management of the disease. Future studies should employ longitudinal designs to track the trends and the underlying causes of hypertension in Bangladesh.

## Figures and Tables

**Table 1:** Sociodemographic and Body Fat Composition Characteristics of Males and Females in Bangladesh.

	Sex of Respondents			
	Females (n = 4215 [53%])	Males (n = 3804 [47%])	Total (n = 8019 [100%])	*p*-Value
	n (100%)	n (100%)	n (100%)	
Characteristics				
Age				<0.001
18–39	2491 (59.1%)	1875 (49.3%)	4366 (54.4%)	
40–59	1509 (35.8%)	1560 (41.0%)	3069 (38.3%)	
60+	215 (5.1%)	369 (9.7%)	584 (7.3%)	
Residence				0.029
Urban	2028 (48.1%)	1885 (49.6%)	3913 (48.8%)	
Rural	2187 (51.9%)	1919 (50.4%)	4106 (51.2%)	
Level of Education				<0.001
No Education	1933 (45.9%)	1718 (45.2%)	3651 (45.5%)	
Primary	1450 (34.4%)	1008 (26.5%)	2458 (30.7%)	
Secondary	615 (14.6%)	745 (19.6%)	1360 (17.0%)	
Tertiary	217 (5.1%)	333 (8.8%)	550 (6.9%)	
Employment				<0.001
Yes	473 (11.2%)	3517 (92.5%)	3990 (49.8%)	
No	3742 (88.8%)	287 (7.5%)	4029 (50.2%)	
Fruits and vegetable intake per day				<0.001
<2 servings	161 (3.8%)	138 (3.6%)	299 (3.7%)	
2–4 servings	2679 (63.6%)	2650 (69.7%)	5329 (66.5%)	
5+ servings	1375 (32.6%)	1016 (26.7%)	2391 (29.8%)	
Physical activities				<0.001
low PA (<600 MET-min/week)	1253 (29.7%)	538 (14.1%)	1791 (22.3%)	
Moderate PA (600–3000 MET-min/week)	2587 (61.4%)	2322 (61.0%)	4909 (61.2%)	
High PA (3000+ MET-min/week)	375 (8.9%)	944 (24.8%)	1319 (16.4%)	
Alcohol Consumption				<0.001
Yes	529 (12.6%)	1572 (41.3%)	2101 (26.2%)	
No	3686 (87.4%)	2232 (58.7%)	5918 (73.8%)	
Tobacco Use				<0.001
Yes	37 (0.9%)	1886 (49.6%)	1923 (24.0%)	
No	4178 (99.1%)	1918 (50.4%)	6096 (76.0%)	
Hypertension				<0.001
Yes	984 (23.3%)	713 (18.7%)	1697 (21.2%)	
No	3231 (76.7%)	3091 (81.3%)	6322 (78.8%)	
Waist Circumference (WC)				<0.001
Low	2179 (51.7%)	3326 (87.4%)	5505 (68.6%)	
Moderate	942 (22.3%)	376 (9.9%)	1318 (16.4%)	
High	1094 (26.0%)	102 (2.7%)	1196 (14.9%)	
Body Mass Index (BMI)				<0.001
Underweight	487 (11.6%)	578 (15.2%)	1065 (13.3%)	
Normal	2157 (51.2%)	2384 (62.7%)	4541 (56.6%)	
Overweight	429 (10.2%)	127 (3.3%)	556 (6.9%)	
Obese	1142 (27.1%)	715 (18.8%)	1857 (23.2%)	
Waist-Hip Ratio (WHR)				<0.001
Low	1268 (30.1%)	2938 (77.2%)	4206 (52.5%)	
Moderate	876 (20.8%)	589 (15.5%)	1465 (18.3%)	
High	2071 (49.1%)	277 (7.3%)	2348 (29.3%)	
Waist-to Height-Ratio (WHtR)				<0.001
Slim	238 (5.6%)	514 (13.5%)	752 (9.4%)	
Normal	1051 (24.9%)	2072 (54.5%)	3123 (38.9%)	
Overweight	1029 (24.4%)	661 (17.4%)	1690 (21.1%)	
Obese	1897 (45.0%)	557 (14.6%)	2454 (30.6%)	

Source: Computed from STEPwise approach to non-communicable disease risk factor surveillance (STEPS) (Bangladesh) 2018. Note: n denotes number of observations; WHtR: Waist-to Height-Ratio; WHR: Waist-Hip Ratio; BMI: Body Mass Index; PA: Physical Activities; WC: Waist Circumference; MET: Metabolic Equivalent Task.

**Table 2:** Prevalence of Blood Pressure, Pulse Rate and Intake of Antihypertension Medications among Males and Females in Bangladesh.

	Sex of Respondents		
	Females (49.50%)	Males (50.40%)	Total (100%)
Categories	%, 95% CI	%, 95% CI	%, 95% CI
Systolic blood pressure			
Normal	24.00, (0.46–0.51)	23.54, (0.44–0.49)	47.54, (0.45–0.50)
Prehypertension	16.78, (0.32–0.36)	19.82, (0.37–0.42)	36.60, (0.34–0.39)
Hypertension	8.72, (0.15–0.20)	7.14, (0.12–0.16)	7.89, (0.14–0.18)
Diastolic blood pressure			
Normal	22.99, (0.44–0.49)	29.87, (0.56–0.62)	27.53, (0.51–0.57)
Prehypertension	14.51, (0.27–0.31)	12.61, (0.23–0.27)	13.62, (0.24–0.29)
Hypertension	12.00, (0.22–0.27)	8.02, (0.14–0.18)	11.02, (0.18–0.23)
Pulse rate			
Abnormal	4.16, (0.07–0.10)	6.74, (0.12–0.15)	5.56, (0.90–0.13)
Normal	45.34, (0.90–0.93)	43.75, (0.85–0.88)	44.69, (0.88–0.91)
Antihypertension medications			
Yes	34.03, (0.66–0.71)	31.5, (0.60–0.65)	65.53, (0.63–0.68)
No	15.48, (0.29–0.34)	19.00, (0.35–0.40)	34.47, (0.33–0.38)

Source: Computed from STEPwise approach to non-communicable disease risk factor surveillance (STEPS) (Bangladesh) 2018. Note: %—percent; 95% CI—95 percent confidence interval.

**Table 3:** Anthropometric, Sociodemographic and Behavioural Factors Associated with Hypertension among Males and Females in Bangladesh.

	Hypertension: a^1^ = No, Yes			
	Females		Males	
	OR, 95% CI	AOR, 95% CI	OR, 95% CI	AOR, 95% CI
Characteristics				
Age				
18–39	a^1^	a^1^	a^1^	a^1^
40–59	2.19, 2.04–2.48 **	3.36, 2.83–3.99 **	2.08, 2.02–2.19 *	2.61, 2.14–3.19 *
60+	2.90, 2.43–3.73 ***	4.71, 3.87–6.27 ***	3.42, 2.65–4.73 ***	3.78, 2.76–5.17 ***
Residence				
Urban	a^1^	a^1^	a^1^	a^1^
Rural	1.04, 1.01–1.09 *	1.12, 1.05–1.22 *	1.10, 1.02–1.18 *	1.19, 1.01–1.37 **
Level of Education				
No Education	a^1^	a^1^	a^1^	a^1^
Primary	0.72, 0.65–0.88 ***	0.77, 0.73–0.96 *	0.91, 0.84–0.98 *	0.86, 0.82–0.91 *
Secondary	0.59, 0.41–0.79 *	0.52, 0.53–0.86 **	0.64, 0.56–0.75 *	0.61, 0.56–0.69 *
Tertiary	0.53, 0.43–0.72 **	0.48, 0.31–0.74 **	0.39, 0.16–0.61 ***	0.42, 0.26–0.64 ***
Fruits and Vegetable Intake per Day				
<2 servings	<2 servings	a^1^	a^1^	a^1^
2–4 servings	2–4 servings	0.58, 0.44–0.72 *	0.45, 0.31–0.71 *	0.61, 0.54–0.76 ***
5+ servings	5+ servings	0.35, 0.26–0.49 **	0.31, 0.23–0.45 *	0.43, 0.35–0.58 ***
Physical Activities				<0.001
low PA (<600 MET-min/week)	a^1^	a^1^	a^1^	a^1^
Moderate PA (600–3000 MET-min/week)	0.56, 0.35–0.82 **	0.69, 0.57–0.95 ***	0.73, 0.59–0.93 ***	0.87, 0.82–0.99 ***
High PA (3000+ MET-min/week)	0.42, 0.33–0.54 ***	0.47, 0.39–0.64 ***	0.51, 0.42–0.66 ***	0.67, 0.62–0.79 ***
Alcohol Consumption				
Yes	a^1^	a^1^	a^1^	a^1^
No	0.41, 0.24–0.68 *	0.39, 0.21–0.63 *	0.55, 0.36–0.74 **	0.53 0.34–0.80 **
Tobacco Use				<0.001
Yes	a^1^	a^1^	a^1^	a^1^
No	0.65, 0.53–0.87 *	0.69, 0.51–0.89 *	0.72, 0.61–0.91 ***	0.78, 0.69–0.93 ***
Waist Circumference (WC)				
Low	a^1^	a^1^	a^1^	a^1^
Moderate	1.72, 1.64–1.91 **	1.96, 1.83–2.48 **	2.14, 2.05–2.28 **	2.03, 2.02–2.13 **
High	2.51, 2.05–2.98 **	2.67, 2.55–2.86 **	2.65, 2.34–2.85 **	2.87, 2.82–2.99 **
Body Mass Index (BMI)				<0.001
Underweight	a^1^	a^1^	a^1^	a^1^
Normal	1.21, 1.13–1.45 *	1.67, 1.49–1.91 *	2.32, 2.11–2.69 **	2.21, 2.15–2.72 **
Overweight	1.35, 1.23–1.62 *	2.05, 1.98–2.18 **	2.40, 2.34–2.62 *	2.49, 1.84–3.20 **
Obese	1.68, 1.44–1.89 ***	2.32, 2.14–2.46 **	2.62, 2.02–2.94 ***	2.73 2.26–3.09 ***
Waist-Hip Ratio (WHR)				
Low	a^1^	a^1^	a^1^	a^1^
Moderate	1.12, 1.04–1.31 **	1.11, 1.02–1.39 *	2.34, 2.29–2.68 *	2.22, 2.01–2.53 *
High	1.39, 1.16–1.79 ***	2.08, 1.45–3.94 **	2.86, 2.45–3.07 *	2.72, 1.81–4.35 *
Waist-to Height-Ratio (WHtR)				<0.001
Slim	a^1^	a^1^	a^1^	a^1^
Normal	0.63, 0.58–0.87 *	0.78, 0.71–0.92 **	0.89, 0.81–0.99 *	0.95, 0.91–1.03
Overweight	1.19, 1.02–1.39 *	1.08, 1.01–2.27 ***	2.32, 2.22–2.81 **	2.22, 2.15–2.45 *
Obese	1.53, 1.28–1.98 **	2.03, 1.82–2.51 ***	2.61, 2.48–3.17 ***	2.56, 2.38–2.89 ***

Source: Computed from STEPwise approach to non-communicable disease risk factor surveillance (STEPS) (Bangladesh) 2018. Note: n denotes number of observations; a^1^ is reference element; * *p* < 0.05, ** *p* < 0.01, *** *p* < 0.001 *t* test statistical significance; WHtR: Waist-to Height-Ratio; WHR: Waist-Hip Ratio; BMI: Body Mass Index; WC: Waist Circumference; AOR adjusted odd ratios, OR: unadjusted odds ratios; PA: Physical Activities; MET: Metabolic Equivalent Task; 95% CI—95 percent confidence interval.

## Data Availability

The data used for the study is available at: https://extranet.who.int/ncdsmicrodata/index.php/access_licensed/track/914. (accessed 25 March 2021).
